# Morphometric Analysis of the Internal Auditory Canal by Computed Tomography Imaging

**DOI:** 10.5812/iranjradiol.7849

**Published:** 2012-06-30

**Authors:** Sergio Ricardo Marques, Sergio Ajzen, Giuseppe D´Ippolito, Luis Alonso, Sadao Isotani, Henrique Lederman

**Affiliations:** 1 Department of Morphology and Genetics, Sao Paulo Federal University, Sao Paulo, Brazil; 2 Department of Diagnosis by Image, Sao Paulo Federal University, Sao Paulo, Brazil; 3 Institutes of Physics, Sao Paulo Federal University, Sao Paulo, Brazil

**Keywords:** Temporal Bone, Inner Ear, Tomography, Morphology, Auditory Canal

## Abstract

**Background:**

Many clinical and experimental studies have been done to analyze the anatomical and functional aspects of the internal auditory canal (IAC) in human beings since there are great inter-individual variability and structural variations that may occur regarding the other adjacent structures.

**Objectives:**

The purpose of this study was to characterize the morphology of the internal auditory canal (IAC) during development using high resolution computed tomography (CT) and to analyze its dimensions, which will be determined by measuring the nearby areas and structures using a system of digital image processing.

**Patients and Methods:**

CT images of the IAC of 110 normal subjects aged 1 to 92 years (mean age, 46.5 years) of both genders were reviewed to determine the shape, area, opening width (OW), longitudinal length (LL), vertical diameter (VD) and distance from the vestibular aqueduct.

**Results:**

The shapes observed in children and adults were funnel-shaped (74% and 58.3%, respectively), cylindrical (22% and 30.9%, respectively) and bud-shaped (4% and 10.8%, respectively). The measurements by CT in children were: area= 50.30 mm^2^, OW = 7.53 mm, length = 11.17 mm, VD = 4.82 mm and the distance between the IAC and the vestibular aqueduct (VA) = 12.63 mm. In adults, the measurements were: area = 44.64 mm^2^, OW = 7.10 mm, length = 9.84 mm, VD = 4.47 mm and the distance between IAC and VA = 11.17 mm.

**Conclusions:**

CT images showed that the IAC has different shapes and when the measurements obtained for children were compared with those of adults, the parameters that presented statistically significant differences in either gender were length and diameter.

## 1. Background

The introduction of computerized transverse axial scanning (tomography) is a milestone in the development of applied radiology (1, 2). Quantitative and morphometric assessment of the internal auditory canal (IAC) are essential to establish the anatomical bases for microsurgery of the cerebellopontine angle and acoustic neuroma, which may produce bone changes and is an important intracranial pathology (3, 4, 5). Determining gender when identifying individuals is still of the utmost importance for assessing the human body, and it is particularly interesting in the fields of forensics, anthropology and archeology. Many clinical and experimental studies have been done to analyze the anatomical and functional aspects of the IAC in human beings; however, with the advance of new diagnostic techniques in the area of otology, studies of the human temporal bone are being redone to provide better anatomical knowledge for surgeons, since there are great interindividual variability and structural variations that may occur regarding the other adjacent structures. These studies will hopefully avoid misinterpretations and improve the quality of radiological results (6, 7, 8). It is essential to know the temporal bone well since it is very complex. Each one of its structures develops differently and they are all very close to each other. According to the literature, the IAC is accessed via the middle cranial fossa. The IAC is a narrow canal that extends for roughly 1cm within the petrous part of the temporal bone and has the shape of an oval foramen. The IAC is closed laterally by a thin and perforated sheet of bone that separates it from the inner ear. It is through this plane that the blood vessels, the facial nerve and the vestibulocochlear nerve pass. This nerve divides into two near the lateral extremity of the internal acoustic meatus, forming the cochlear nerve and the vestibular nerve (9). Since previous reports indicate variation in the anteroposterior (AP) diameter and length of the canal, in order to avoid damages to the labyrinth, a preview of the measurements of the IAC is important for interpreting radiographs (10). Variations between the left and right sides are also very common. The canal is considered stenotic if its diameter is smaller than 2 mm. The normal diameter varies from 4 to 8 mm (11, 12). There are many publications that describe how the shape, size and position of the human IAC can influence certain inner ear disorders. The IAC may serve as a canal for inner ear infection spreading that could damage it or reach the central auditory pathways (9, 13, 14). There are many techniques that can be used to determine the location of the IAC. Among them high resolution CT is a sensitive method for the detection of anomalies that allows the correlation between radiological images and clinical data. This canal is better seen in axial views (6, 14-20).

## 2. Objectives

The purpose of this study was to characterize the morphology of the IAC (IAC) in children and adults using high resolution CT (CT) and to analyze its dimensions, which will be determined by measuring the nearby areas and structures using a system of digital image processing.

## 3. Patients and Methods

A total of 110 subjects were retrospectively investigated. Their age ranged from 1 to 92 years. There were 28 men, 26 boys, 32 women and 24 girls. CT scan study of the temporal bone was performed for all the patients due to clinical problems such as headache, tinnitus, hearing loss, mild acute recurrent otitis and other symptoms of unknown non-congenital and non-anatomical etiology. CT images of the temporal bones of these patients who had undergone routine tests were obtained from the Department of Diagnostic Imaging of Sao Paulo Federal University, Brazil. The project was approved by the Research Ethics Committee of Sao Paulo Federal University/Hospital (protocol number 1626/07). The tomography scanner Philips Secura™ was used for obtaining the images. The protocol included taking shots from the axial plane with the patients lying on their back and from the supraorbitomeatal plane with a head support specific for this position. Axial cuts in the supraorbitomeatal plane were assessed (horizontal) where the vestibular aqueduct and the internal acoustic meatus were shown with the patients in the dorsal decubitus position. The cuts were 1.0 mm wide and taken at 1.0 mm intervals. The images were acquired by a high resolution algorithm with parameters of 120 Kvp, 180mAs, exposure time of 1.00s and a matrix of 512×512.

**Figure 1:**
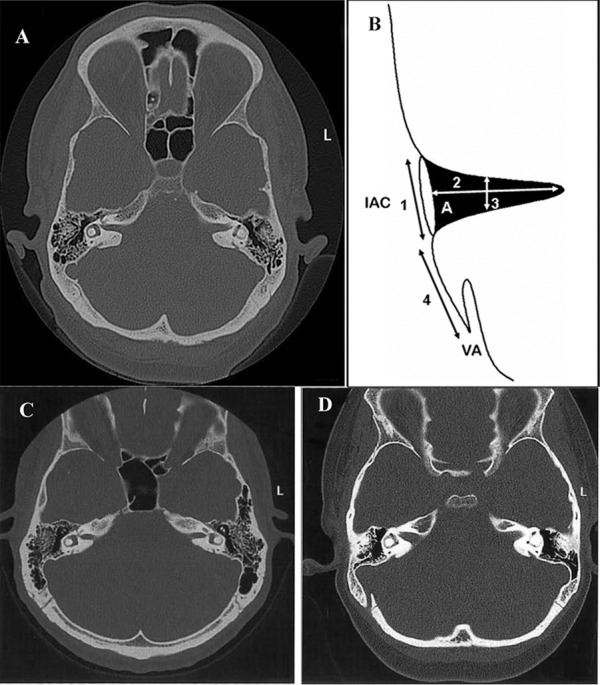
A. Computed axial tomography image of the temporal bones of a 58-year-old female patient. B. Diaphragm of the IAC, illustrating the reference points for the measurements: (A) – area; (1) opening width; (2) longitudinal length, the opening of the canal until its end along its axis; (3) AP diameter of the total length of this canal; (4) the distance between the openings of the IAC and the vestibular aqueduct. C. Computed axial tomography images of the temporal bones of a 29-year-old male patient and an 8-year-old female patient (D)

The following features were measured: opening width, longitudinal length from the opening to the end of the canal along its axis, AP diameter of the length of the canal, distance between the openings of the IAC and vestibular aqueduct, and selected area of the IAC (Figure 1). The shapes of the IAC were defined as cylindrical, funnel-shaped or bud-shaped. More detailed ways for measurements were as follows: (1) The distance from the more concave part of the posterior lip of the IAC wall to the more medial border of its anterior wall was used for measuring the opening width of the IAC; (2) The distance that goes from the midpoint of the IAC to the extremity of the canal in its most concave portion was used for measuring the length of the IAC; (3) A perpendicular straight line drawn from the midpoint of the IAC length uniting the anterior and posterior walls was used for measuring the AP diameter; (4) The distance between the more concave wall of the posterior lip of the IAC to the medial border of the vestibular aqueduct was used for measuring the distance between the IAC to the vestibular aqueduct; (5) The entire region between the points used for measuring the opening of the IAC to its more concave extremity was used for calculating the area. A computer program named SPCIM (Image Processing System) developed by the Institute of Physics of Sao Paulo Federal University, called Computed Image Processing System, was used for analyzing the images (18). The software was calibrated in a way that standardized the size of all images. After this procedure, calculations of the marked area, diameter, distance, length and opening began. The software only recognized the area inside the loop and the selected measurement points. It calculated these points through the number of pixels (i = i + 1) that composed the image and thus the matrix, in a linear unit of area (mm^2^) and length (mm). Regarding the intraobserver reliability, the measurements were made by the same tester with an interval of three days between measurements. The anatomical points are well delinated and visible in the images. We have obtained great intraobserver reliability based on the acquisition of the intraclass correlation coefficient, which exceeded 0.75.

The final data were divided into two groups: children up to 14 years of age and adults aged 19 to 92 years. These groups were then assessed by the Wilcoxon and Mann-Whitney tests. The significance level was set at P ≤ 0.05 and the data analysis was carried out using SPSS 16 for Windows™ (SPSS Inc., Chicago, IL). Only the temporal bone structures of patients who presented anatomical integrity of temporal bone and craniofacial compartment were used. Structures that presented any type of trauma, tumor lesions, inflammatory processes, indication of surgical manipulation and any acquired or congenital anatomical anomalies in this region were excluded.

## 4. Results

**Figure 2:**
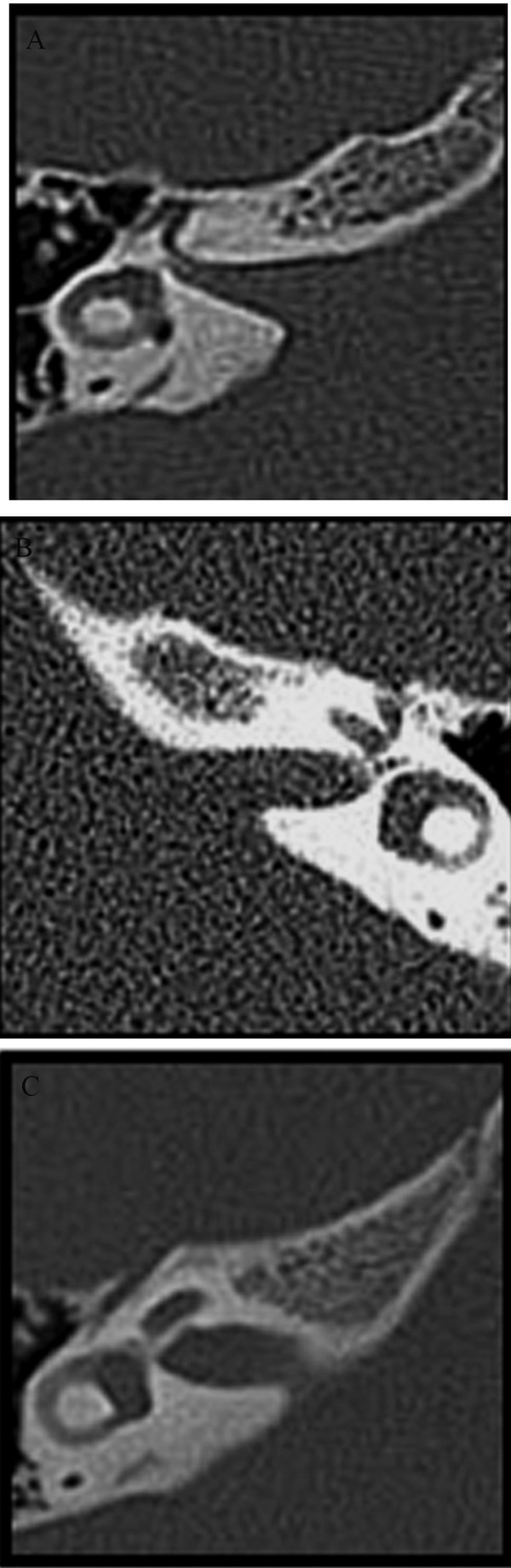
Types of shapes observed for the IAC in CT images A) Funnel-shaped, B) Cylinder-shaped, C) Bud-shaped

**Figure 3:**
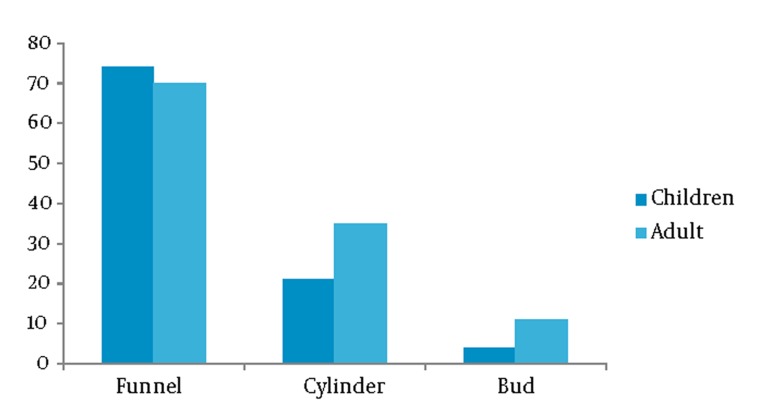
Shape distribution of the IAC of 50 children (100 temporal bones) and 60 adults (120 temporal bones) of both genders observed by CT

The variations in the shapes of the human IAC seen in the CT images of adults and children were classified into three types: funnel-shaped, cylindrical and bud-shaped (Figure 2). The shapes of the IAC vary. In adults, the most frequent shape was the funnel-shaped (58.3%), followed by the cylindrical (30.9%) and finally the bud-shaped (10.8%). Although the order remained the same in children, the distribution varied: 74% were funnel-shaped, 22% were cylindrical and 4% were bud-shaped (Figure 3), which shows that the radiographic aspect usually depends on the orientation of the canal in relation to the tomographic cut plane. In children, the bilateral measurements of the IAC resulted in a mean area of 50.30 mm^2^, mean opening width of 7.53mm, mean length of 11.17 mm, mean AP diameter of 4.82 mm and mean distance between the IAC and the vestibular aqueduct of 12.63 mm (Table 1). The areas, external opening widths and distances did not differ significantly between sides or between genders, but there were statistical differences in diameter and length when compared with those of adults. In adults, the bilateral measurements of the IAC resulted in a mean area of 44.64 mm^2^, mean opening width of 7.10 mm, mean length of 9.84 mm, mean AP diameter of 4.47 mm and mean distance between the IAC and the vestibular aqueduct of 11.17 mm (Table 1). The areas, external opening widths and distances did not differ significantly between sides or between genders, but there were statistical differences in diameter and length when compared with those of children.

**Table 1:** Measurements of the IAC Observed by Computed Tomography

	**Area IAC, mm^2^**	**Opening Width IAC** ^a^**, mm**	**Length IAC, mm**	**AP Diameter IAC, mm**	**Distance IAC/VA** ^a^**, mm**
Children, No. (min ^a^-max ^a^) SD ^a^	50 (47.9-52.6) ± 3.10	50 (7.59-7.48) ± 0.20	50 (11.05-11.29) ± 0.22	50 (4.64-5.00) ± 0.13	50 (12.31-12.96) ± 0.51
Total mean	50.30	7.53	11.17	4.82	12.63
Adults, No. (min ^a^ -max ^a^) SD ^a^	60 (49.3-39.9) ± 3.54	60 (7.43-6.76) ± 0.16	60 (10.2-9.43) ± 0.22	60 (4.48-4.46) ± 0.10	60 (11.6-10.7) ± 0.55
Total mean	44.64	7.10	9.84	4.47	11.17

^a^Abbreviations: IAC, internal auditory canal; Max, maximum; Min, minimum; SD, standard deviation; VA, vestibular aqueduct

## 5. Discussion

In the literature review, the IAC presents different shapes, positions and sizes. A Greek study from 1972 (21) analyzed 242 human temporal bones of individuals aged 16 to 93 years and found intra-individual shape variations (right and left sides). The same group, in 1975, (22) reanalyzed the same sample, although this time stratifying the sample by age and gender. No significant differences were found regarding shape. Yet, the values found by our study are very different from those of the two studies mentioned above. It is likely that our results are due to the racial heterogeneity of our sample, since there is much racial miscegenation in the Brazilian population: individuals with black, white and yellow ancestry are very common. Considering that anatomical elements are determined during embryogenesis and that the related attribute is inherited as a polygenic trait (where many genes contribute with additive effects), there is a diversity among these patterns in different races and ethnic groups. Thus, such differences may explain the divergence between our results and those obtained from a more homogeneous racial sample. Silicone casts in 30 paired temporal bones showed that 16.7% of them are straight, 43.3% of them are medially narrow and 26.7% of them are oval (23). Since the types were different, there was no comparison between methodologies. According to the standardized criteria for polytomography classification, the internal acoustic meatus of 115 patients of the normal group, of which 63 were males with a mean age of 38 years (range, 11 to 74 years) and 52 were females with a mean age of 40 years (range, 13 to 83 years) were as follows: 70% straight, 14% medially narrow and 14% oval (4, 24). According to the same criteria and methodology used in 300 normal subjects where 180 were male and 120 were female (mean age, 47 years; range, 14-77 years), the shape of the internal acoustic meatus was cylindrical in 436 ears (72.7%), bud-shaped in 137 ears (22.8%) and funnel-shaped in 27 ears (4.5%) (15, 16). In this study, the funnel shape IAC was the most common in both children and adults (74% and 58.3%, respectively), followed by the cylindrical shape (22% and 30.9%, respectively) and the bud shape (4% and 10.8%, respectively). Therefore, the funnel shape prevailed in children and adults, not confirming the findings of the above mentioned authors. CT of 125 patients with otosclerosis and polytomography of 175 patients with other conditions, totalling 300 patients, revealed that 51% of the IACs were straight, 30% were medially narrow and 19% were oval (12). Since their morphological classification differed from that of this study, it was not possible to compare both studies. Measurements of the IAC for gender determination are still frequently discussed in the literature, but remain controversial. Using a surgical microscope, 12 cerebellopontine angles were dissected from adult cadavers from both genders and measured with a caliper. Their mean area was 139.09 mm^2^ (86.45- 196.04 mm^2^) (3). This value is much higher than the values found in this study, which were 50.30 mm^2^ in children and 44.64 mm^2^ in adults. The authors mentioned the below made measurements similar to those made by this study and obtained similar results for children and adults: opening width of 7.53 mm and 7.10 mm respectively, length of 11.17 mm and 9.84 mm, respectively, AP diameter of 4.82 mm and 4.47 mm, respectively and distance between the canal and the vestibular aqueduct of 12.63 mm and 11.17 mm, respectively. Fujita and Sando (10) used a software to measure the length and the distance between the canal and vestibular aqueduct in 10 temporal bones of cadavers aged 4 months to 70 years and found that the length ranged from 5.9 to 11.7 mm and the distance ranged from 6.2 to 12 mm. They also measured the length and AP diameter in 108 temporal bones of adults of both genders and found them to be 11.5 mm and 3.6 mm, respectively (25). These numbers are in agreement with the study of 20 dissected human temporal bones, which resulted in a length of 10.1 mm (26). In 242 samples from individuals aged 16 to 93 years, the opening width was 4.5 mm (3-7 mm), the AP diameter was 4.6 mm (2-7 mm) and the length was 9.2 mm (6-14 mm). The same author using the same sample found AP diameters from 4.0 to 5.0mm and lengths from 8.0 to 11mm (21-22mm). In 108 temporal bones, the AP diameter was 4mm (2-6 mm) and the length was 8.5 mm (5-14 mm) (27). In 14 temporal bones of individuals aged 35 to 60 years, the AP diameter was 4.0 mm (3-5 mm), the length was 8.4 mm (7-11 mm) and the opening width was 5.0 mm (3-7 mm) (28). In 90 temporal bones of individuals of both genders, the length was 10.98 mm (8-15 mm) (29). In 180 temporal bones, the AP diameter was 4.0 mm (2-6 mm) and length was 8.5 mm (3-16 mm).(27)

Using the same methodology in temporal bones separated by gender, Lang (30) found a length of 11.15 mm, an AP diameter ranging from 3.0 to 7.0 mm, and an opening width of 6.46 mm. In children, Lang reported a 7.23 mm length, a 4.6 mm AP diameter and a 4.3 mm opening width. Our study found a slightly greater length and opening width in children. Escajadillo (31) studied 50 temporal bones of individuals aged 18 to 70 years and found a 6.9mm (4-10 mm) canal length, which is much smaller than the value found in this study. Analysis of histological cuts of the temporal bones of 435 normal patients resulted in an AP diameter of 3.7 mm (2-6 mm) and an opening width of 3.7 mm (2-5 mm), six values that are much smaller than those found by this study. Using silicone casts in 30 temporal bones, Amjad et al. (23) found a length of 9.9 mm (8-13 mm) and an AP diameter of 5.9 mm (4-8 mm). Despite the criteria used, the values were similar and greater than those found in our sample. The values found by the following authors who used CT images for the measurements found values very close to those of the present study. In 21 CT studies, the length was 10.4 mm (7-15 mm) (26). In 20 temporal bones of individuals aged 1 to 72 years, of both genders, the length found by tridimensional reconstruction was 10.6 mm (6.9-14.1 mm) on the upper wall and 9.6 mm (4.5-13.7 mm) on the lower wall (32). When the measurements were made by software in 54 patients, the length was 11.6 mm (8.5-16.5 mm) (33). CT can measure the IAC efficiently. The canal can be seen best when the axial perspective is used (5, 18, 20, 33-35). Polytomography in 125 patients with otosclerosis and 175 patients with other conditions revealed a length of 8 mm (4-11 mm) and a AP diameter of 4 mm (2-8 mm) (12). In 100 normal skulls, the opening width was 5.7 mm (4-8 mm) (34). In 250 temporal bones, the opening width was 6.2 mm, the AP diameter was 5.2 mm (2.5-11 mm) and the length was 7.8mm (3-16 mm) (4). Only the last parameter had a value below that found in our study. Computed axial tomography of 50 normal individuals aged 19 to 79 years of both genders revealed an opening width ranging from 5.0 to 7.0 mm and a length ranging from 6.0 to 11.0 mm (5). In another study of 110 patients aged 1 to 92 years also using software, the distance between the canal and the vestibular aqueduct was 11.35 mm in adults and 11.88 mm in children (18). These values are similar to those found in this study for both genders. A study of 309 temporal bones of children aged 2 months to 15 years of both genders, using computed axial tomography found an opening width of 5.02 mm (19). Another study using the same technique in 32 temporal bones found an opening width of 5.9 mm (4-8.5 mm) (36). Our values for the opening width are greater than those; 7.53 mm for children and 7.10 mm for adults. Computed axial tomography was used to study normal variations in 650 human temporal bones from individuals aged 4 months to 78 years, which revealed three cases of an AP diameter of 8 mm (37). The same procedure was used to study 25 patients aged 4 months to 36 years without sensorineural hearing loss; the opening width was 5.3 mm (4-8 mm), the length was 9.5 mm (7-13 mm) and the AP diameter was 6.1 mm (4-9 mm). A small variation in the dimensions does not interfere with normal hearing (38) and can be symmetrical in any individual (39). The AP diameter found by these authors is much greater than that found in this study. Computed axial tomography was used in 67 normal subjects aged 18 to 83 years, revealing an opening width of 7.39 mm on the right side and 7.49 mm on the left side, an AP diameter of 4.43 mm on the right side and 4.55 mm on the left side, and a length of 11.34 mm in the right ear and 11.33 mm in the left ear (17). These values are similar to those found by our study. Ninety-seven dissected specimens of temporal bones investigated with computed axial tomography revealed an AP diameter of 4.22 mm (2.3-6.8 mm) and a length of 11.31 mm (6.2-14.8 mm) (35). These values are similar to those of the present study. The length of the IACs of children and adults differed significantly; the lengths in children are significantly greater than those in adults. Meanwhile, the diameters found in children are significantly greater than that found in adults. The area found for the two groups does not differ statistically. Therefore, it is possible for greater lengths and smaller diameters to occur during childhood and as the individual develops, there is a gradual shortening of the length and an increase of the diameter, which does not change the total area of the canal. A morphometric analysis by CT of the IAC of five patients aged 5 to 37 years found an AP diameter of 1.8 mm (1.4-2.2 mm) and a length of 14 mm (12-17 mm) (40). CT of seven fresh, dissected cadavers revealed an AP diameter of 2.31 mm (0.65-7.5 mm) (41). The values found for AP diameter are much lower than those found in this study, while the values for length are greater. A study analyzed 25 disarticulated temporal bones and 58 articulated temporal bones (42) of adults of unknown gender, measuring the distance between the IAC and the vestibular aqueduct with a digital caliper. The distance was 9.89 mm, which is similar to that found by the present study and which validates direct measurement as one of the techniques that can be used for this purpose. An anatomical and radiological study using CT to investigate the relationships between the IAC and other structures in 59 temporal bones found a length of 8.4 mm.8 Another study using CT in 115 patients aged 13 to 83 years of both genders, found an AP diameter of 5.1 mm (3-8 mm) and a length of 7.1 mm (3-11 mm) (24). Finally, a study also using CT in 300 patients aged 15 to 77 years found a length of 8.7 mm (4-18 mm) (15). Our study found a much greater length than that found by the authors above.

There are still other possibilities for studying the temporal bone, such as measuring the lateral angle of the IAC, which may prove useful for gender determination (43). This forensic strategy was not used in the present study, but can contribute to the measurements used herein to improve the characterization of this skull compartment. In conclusion, the shape of the IAC varies greatly. There are no statistical differences between the areas, external opening widths and distances between the IAC and vestibular aqueducts between children and adults of both genders. However, there are statistical differences between its length in adults and children and its AP diameter. A thorough knowledge of the normal anatomy of the temporal bone and the anomalies that affect it are important for interpreting radiographs, as it improves the quality of the results and allows development of a new diagnostic criterion.
